# Induction Heating Triggers Antibiotic Release and Synergistic Bacterial Killing on Polymer‐Coated Titanium Surfaces

**DOI:** 10.1002/adhm.202202807

**Published:** 2023-05-01

**Authors:** Jan C. Kwan, Ronald S. Flannagan, Mónica Vásquez Peña, David E. Heinrichs, David W. Holdsworth, Elizabeth R. Gillies

**Affiliations:** ^1^ School of Biomedical Engineering The University of Western Ontario 1151 Richmond Street London Ontario N6A 5B9 Canada; ^2^ Bone and Joint Institute The University of Western Ontario The Sandy Kirkley Centre for Musculoskeletal Research University Hospital B6‐200 London Ontario N6G 2V4 Canada; ^3^ Department of Microbiology and Immunology The University of Western Ontario 1151 Richmond Street London Ontario N6A 5C1 Canada; ^4^ Imaging Research Laboratories Robarts Research Institute The University of Western Ontario 1151 Richmond Street London Ontario N6A 2B8 Canada; ^5^ Department of Medical Biophysics The University of Western Ontario 1151 Richmond Street London Ontario N6A 5C1 Canada; ^6^ Department of Chemistry The University of Western Ontario 1151 Richmond Street London Ontario N6A 5B7 Canada; ^7^ Department of Chemical and Biochemical Engineering The University of Western Ontario 1151 Richmond Street London Ontario N6A 5B9 Canada

**Keywords:** antibacterials, biofilms, coatings, induction heating, orthopedic infections, poly(ester amide)

## Abstract

Infection is a major complication associated with orthopedic implants. It often involves the development of biofilms on metal substrates, which act as barriers to the host's immune system and systemic antibiotic treatment. The current standard of treatment is revision surgery, often involving the delivery of antibiotics through incorporation into bone cements. However, these materials exhibit sub‐optimal antibiotic release kinetics and revision surgeries have drawbacks of high cost and recovery time. Herein, a new approach is presented using induction heating of a metal substrate, combined with an antibiotic‐loaded poly(ester amide) coating undergoing a glass transition just above physiological temperature to enable thermally triggered antibiotic release. At normal physiological temperature, the coating provides a rifampicin depot for >100 days, while heating of the coating accelerates drug release, with >20% release over a 1‐h induction heating cycle. Induction heating or antibiotic‐loaded coating alone each reduce *Staphylococcus aureus* (*S. aureus*) viability and biofilm formation on Ti, but the combination causes synergistic killing of *S. aureus* as measured by crystal violet staining, determination of bacterial viability (>99.9% reduction), and fluorescence microscopy of bacteria on surfaces. Overall, these materials provide a promising platform enabling externally triggered antibiotic release to prevent and/or treat bacterial colonization of implants.

## Graphical abstract

Orthopedic infections frequently involve bacterial biofilms on metallic implant surfaces. They are very challenging to eradicate. Presented here is a new approach using a combination of induction heating and a thermo‐responsive polymer coating, to enable externally triggered release of antibiotics right at the site of the biofilm.

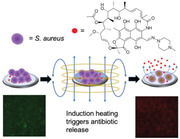

## 1 Introduction

Infection has become one of the major complications associated with orthopedic implants, such as hip and knee replacements, as well as trauma‐related devices including nails, screws, and plates.^[^ ^1^ ^]^ It is estimated that infections occur after surgery for open fractures in up to 40% of cases.^[^ ^2^ ^]^ While the rates of peri‐prosthetic joint infection are lower, at 1–2%,^[^ ^3^ ^]^ it remains a serious complication, and with the number of total hip and knee replacement procedures growing each year, the cost for treatment is estimated to reach 1.85 billion $US per year by 2030.^[^ ^4^ ^]^ Infection can occur through various mechanisms such as hospital‐acquired infections, bacterial contamination of the surgical site, or bacteria entering the surgical site from other locations in the patient's body.^[^ ^1^, ^5^ ^]^ Orthopedic device‐related infection is often associated with the metallic surface of the implant.^[^ ^1b^ ^]^ Planktonic bacteria can interact with the surface, initially through reversible adherence, but then subsequently undergo irreversible attachment and initiate biofilm development.^[^ ^6^ ^]^ Fully mature biofilms act as a barrier to the ability of the host's immune system to clear the infection, and the bacteria growing within biofilms are frequently recalcitrant to antimicrobial therapy.^[^ ^7^ ^]^ In particular, infections caused by *Staphylococcus aureus* (*S. aureus*), are the leading cause of implant failure.^[^ ^8^ ^]^

The current standard of treatment for orthopedic device‐related infection is revision surgery.^[^ ^1b^ ^]^ Systemic antibiotic therapy can accompany revision surgery but it is generally ineffective as a stand‐alone treatment due to the limited ability of antibiotics to penetrate biofilms and kill biofilm bacteria.^[^ ^9^ ^]^ To enhance the efficacy of revision surgery, a two‐stage approach is often used, where antibiotics are loaded into temporary poly(methyl methacrylate) (PMMA) bone cement‐based spacers.^[^ ^10^ ^]^ After the infection is resolved, the second surgery is performed to install the permanent implant. However, PMMA is not biodegradable, and therefore a substantial portion of the antibiotic in the interior of the material is not released.^[^ ^11^ ^]^ It has been reported that the eluted antibiotic concentrations are therapeutic only in the first few post‐operative hours, followed by the release of sub‐inhibitory antibiotic concentrations thereafter.^[^ ^12^ ^]^ This limitation has motivated a shift towards the use of resorbable materials such as calcium sulfate and calcium phosphate, which can degrade over 4–13 weeks in vivo.^[^ ^13^ ^]^ These carriers typically release their entire antibiotic load with an initial burst in the first several days, leaving minimal drug to combat any subsequent post‐surgical infection.^[^ ^14^ ^]^ Thus, new delivery systems with better controlled and prolonged release of antibiotics are needed to treat orthopedic device‐related infection. Moreover, avoidance of revision surgeries is highly desirable as they are more costly, require longer hospitalization, delay recovery, and come with greater risk of re‐infection as compared to primary surgeries.^[^ ^1^, ^15^ ^]^

In recent years, induction heating of metallic implants has been proposed as an approach for the non‐invasive treatment of implant‐related infections through the thermal destruction of biofilms.^[^ ^16^ ^]^ Induction heating involves the use of a high frequency alternating magnetic field (AMF), to generate an electric current (eddy current) within a conductive implanted material such as Ti or steel.^[^ ^17^ ^]^ The flow of the eddy current through the resistance of the material results in rapid Joule heating without any external heat source or contact with the heating device and with minimal heating of surrounding tissues.^[^ ^16^, ^18^ ^]^ Despite this, induction heating alone is unlikely to be sufficient to eradicate infection because even at high temperatures that range from 80–90 °C, viable bacteria were still present on the surface.^[^ ^16^, ^18^, ^19^ ^]^ Moreover, the time and intensity of the heating treatment must be tightly controlled to minimize tissue necrosis. It has been shown that induction heating can potentially be combined with antibiotics to achieve enhanced killing of biofilm bacteria.^[^ ^16^, ^20^ ^]^ However, without an effective means to locally deliver the antibiotics, this approach would still suffer from many of the limitations of systemically administered antibiotics.^[^ ^9^ ^]^

**Figure 1:**
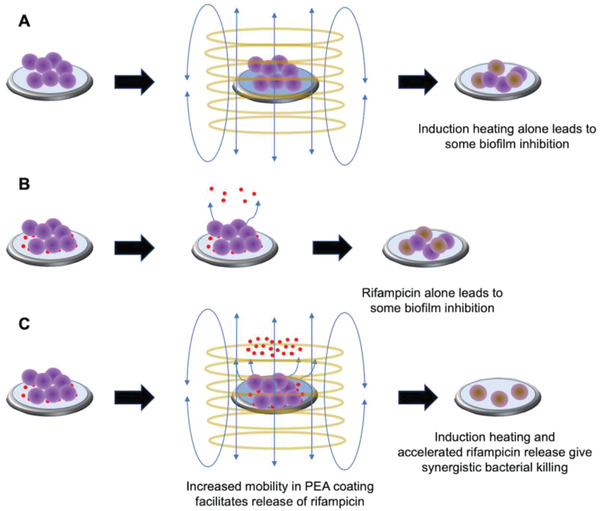
a‐c) Schematic showing an antibiotic‐loaded PEA coating on a Ti disc. Induction heating of the metal disc above the polymer's *T*_g_ triggers the release of antibiotic.

Described here is a new approach for the treatment of orthopedic device‐related infection that combines induction heating with an antibiotic‐loaded polymer coating having a glass transition temperature (*T*_g_) just above physiological temperature. Externally controlled induction heating of the metallic substrate triggers a change in polymer properties as it passes through the *T*_g,_ providing on‐demand antibiotic release that localizes to the site of the biofilm bacteria (**Figure** **1**). While several systems have been demonstrated to provide thermo‐responsive release of antibiotics, they were based on lower critical solution temperature transitions rather than *T*_g_, and exhibited high background drug release rates at the normal physiological temperature of 37 °C.^[^ ^21^ ^]^ Polymeric implant materials composed of poly(butyl methacrylate*‐stat*‐methyl methacrylate) with embedded superparamagnetic iron oxide nanoparticles having *a T*_g_ of 52 °C, were shown to exhibit triggered release of ibuprofen in response to alternating magnetic fields.^[^ ^22^ ^]^ However, to the best of our knowledge, there are no examples of polymeric coatings that have been combined with induction heating for the release of antibiotics. The selected polymer for the current work is a poly(ester amide) (PEA), as PEAs have been well tolerated in vivo^[^ ^23^ ^]^ and their properties can be readily tuned based on their monomer composition.^[^ ^24^ ^]^ Rifampicin was selected as a model antibiotic drug as it is commonly used in the treatment of staphylococcal prosthetic joint infection.^[^ ^25^ ^]^ We demonstrate that in the absence of direct or induction heating, the PEA coating on a Ti substrate erodes slowly, providing an antibiotic reservoir for more than 100 days, while heating the PEA coating above its *T*_g_ triggers an accelerated release of antibiotics. Compared to a rifampicin‐loaded PEA coating or induction heating alone, we show that induction heating of the drug‐loaded coating leads to near complete eradication of biofilm bacteria through synergistic reduction in bacterial viability and biofilm formation. Our approach thereby provides a novel platform to potentially prevent and non‐invasively treat implant‐related infections.

## 2 Results and Discussion

### 2.1 Preparation and Characterization of PEA Coatings

**Figure 2:**
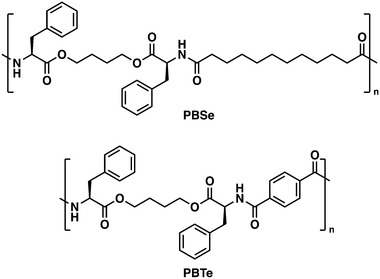
Chemical structures of PBSe and PBTe.

A PEA composed of L‐phenylalanine, 1,4‐butanediol, and sebacic acid (PBSe, **Figure** **2**) was selected as the PEA of interest because it has previously been reported to have a *T*_g_ of about 39 °C.^[^ ^26^ ^]^ As a control polymer to evaluate the effect of heating without passing through a *T*_g_, a different PEA based on L‐phenylalanine, 1,4‐butanediol, and terephthalic acid (PBTe) was chosen (Figure 2). PBTe has been reported to have a *T*_g_ of about 100 °C.^[^ ^24c^ ^]^ PBSe and PBTe were prepared as previously reported.^[^ ^24^, ^26^ ^]^ Titanium alloy Ti6Al4V (Ti) discs (24 mm diameter, 3 mm thickness) were prepared by 3D printing via laser powder bed fusion (LPBF) and then smoothed using hand polishing with tungsten‐carbide burs and finished with shot blasting using ceramic beads. Mean surface roughness (Ra) for similarly prepared disks was measured using surface profilometry in a previous study^[^ ^27^ ^]^ and found to range between 1.7 and 2.6 µm. To prepare the PEA‐rifampicin blend coatings, a solution of PEA and rifampicin was drop cast onto the Ti discs and then dried in vacuo. The resulting coatings were 8.4 ± 1.3 µm thick. While the initial Ti disc had a contact angle of 30 ± 7°, coating with the PEA increased the contact angle to 78 ± 2°. There were no significant changes upon rifampicin incorporation, with the coating containing 5% (w/w) of drug having a contact angle of 77 ± 7°. Scanning electron microscopy showed that there were no substantial changes in the surface features upon coating (Figure S4, Supporting Information). In addition, the coatings adhered well to the underlying substrate, with no delamination observed in solution over 100 days (Figure S5, Supporting Information).

**Figure 3:**
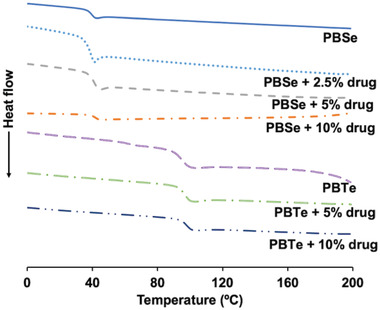
DSC thermographs of pure PBSe and PBTe and their formulations with rifampicin. The incorporation of antibiotic led to modest increases in the *T*_g_.

The thermal properties of the rifampicin‐PEA blend coatings were evaluated by differential scanning calorimetry (DSC) and were found to vary within a few degrees after incorporation of the drug. While PBSe had a *T*_g_ of 39 °C, the incorporation of 2.5, 5 and 10% (w/w) of rifampicin relative to polymer resulted in small increases in the *T*_g_ to 41, 43, and 45 °C, respectively (**Figure** **3**). The incorporation of 5 and 10% (w/w) of rifampicin into the control polymer PBTe resulted in an increase in *T*_g_ from 95 °C for the pure polymer to 97 and 98 °C for 5% and 10% rifampicin respectively. Rifampicin can exist in an amorphous state or in various crystalline forms with melting points between 170 and 240 °C.^[^ ^28^ ^]^ However, there was no evidence of crystalline rifampicin in the blends with the PEA.

### 2.2 In Vitro Release of Rifampicin in the Absence of the Thermal Triggering

**Figure 4:**
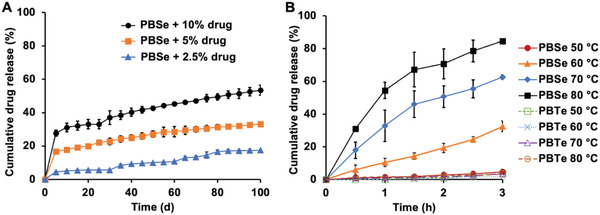
Release of rifampicin from PEA coatings: A) PBSe coatings containing 2.5%, 5%, or 10% (w/w) at 37 °C in pH 7.4 PBS; B) PBSe and PBTe coatings containing 5% (w/w) rifampicin at elevated temperatures in pH 7.4 PBS. More rapid release of drug occurred for the PBSe coatings but not for the PBTe coatings at elevated temperatures. Error bars correspond to the standard deviations (*n* = 3).

First, the release of rifampicin at a physiological temperature of 37 °C from PEA coatings on Ti discs immersed in pH 7.4 PBS was studied (**Figure** **4**). Each of the 2.5, 5 and 10% (w/w) rifampicin‐PBSe coatings underwent an initial burst release, ranging from around 5% release for the 2.5% rifampicin coatings to almost 30% release for the 10% coating, within the first 5 days. This initial release likely corresponded to the drug that was at or near the surface. Subsequently, a slower release occurred, with about 17% of rifampicin released from the 2.5% coating and 53% released from the 10% coating over 100 days. These results indicate that sustained release of rifampicin from PEA coatings can be achieved, providing a depot of remaining drug that can potentially be triggered for release for multiple months after implantation in the event that an infection arises. The influence of pH was also examined, as infection and inflammation can result in a reduction in pH of surrounding tissues. The release rate at pH 5.0 was very similar to that at pH 7.4, with 16% released over the first 20 days for the coating containing 5% rifampicin (Figure S7, Supporting Information).

**Figure 5:**
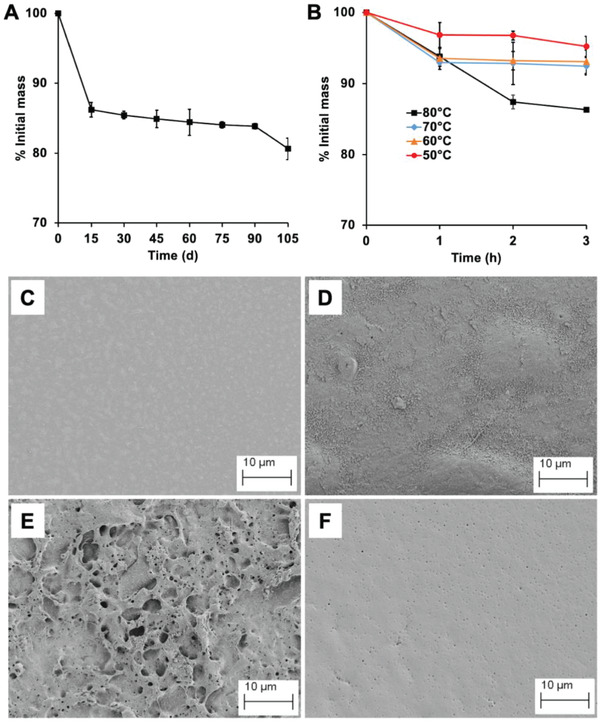
Degradation of PBSe coatings containing 5% (w/w) of rifampicin: A) Mass loss of coatings incubated in pH 7.4 PBS at 37 °C for 105 days; B) Mass loss of coatings incubated at 50–80 °C for 3 h. Scanning electron micrographs C) Prior to incubation; D) After incubation at 37 °C for 60 days; E) After incubation at 37 °C for 105 days; F) After incubation at 80 °C for 3 h. Additional SEM images are included in Figure S8 (Supporting Information). Error bars in (A) and (B) correspond to the standard deviations (*n* = 3).

The slow degradation of the PBSe coatings was also demonstrated by mass loss and scanning electron microscopy (SEM) for 5% (w/w) rifampicin coatings incubated at 37 °C for more than 100 days in PBS. A 15% reduction in coating mass was observed over 15 days (**Figure** **5**A). During the next 90 days, only a further 5% mass loss occurred, confirming the slow degradation of PBSe. SEM of PBSe‐rifampicin coatings, before incubation, showed that the surface of the coating was relatively smooth (Figure 5C). After 60 days, the surface appeared to become rougher, suggesting that some erosion of the polymer surface had occurred (Figure 5D). After 105 days, substantial erosion of the surface was observed (Figure 5E).

### 2.3 Thermally Triggered Rifampicin Release

Next, the ability to trigger the accelerated release of rifampicin above the *T*_g_ of the PEA was demonstrated. The rifampicin‐PEA coatings were incubated in PBS at temperatures from 50–80 °C. At 80 °C, more than 70% of rifampicin was released from the 5% coating (Figure 4B) while almost 90% of the rifampicin was released from the 10% (w/w) rifampicin coating within 3 h (Figure S9, Supporting Information). Even at 60 °C, more than 30% of the rifampicin was released from both the 5% and 10% coatings, with the release slower at 50 °C. Most physicochemical processes, including drug release, are expected to be faster at higher temperatures. Therefore, to demonstrate that the accelerated release of rifampicin from the PBSe coatings was indeed due to the *T*_g_ of the polymer, the release rates of rifampicin from the control PBTe coatings with *T*_g_ values of ≈100 °C were also evaluated at 50–80 °C. At each temperature, 10% or less of the loaded rifampicin was released from these coatings, thereby demonstrating the important role of the PBSe glass transition in facilitating drug release. We also examined the potential of the PBSe coatings to simultaneously encapsulate and release multiple drugs as combination therapy is one approach to address infections involving drug‐resistant bacterial strains.^[^ ^29^ ^]^ Indeed a coating containing 5% (w/w) of each of rifampicin and vancomycin exhibited temperature‐dependent drug release (Figure S10, Supporting Information).

Furthermore, to confirm that the accelerated drug release from the PBSe coatings at elevated temperatures was not simply a result of accelerated PEA degradation, mass loss from the coatings was examined. The mass loss ranged from 5–14% over 3 h from 50–80 °C (Figure 5B). Compared to 37 °C over 100 days, there was more drug release but less mass loss from the PBSe coatings at 50–80 °C over 3 h, confirming that the drug release was not rate‐limited by polymer degradation at the elevated temperatures. Furthermore, SEM micrographs of the surface of PBSe‐rifampicin coatings incubated at 80 °C for 3 h showed minimal polymer degradation but small pores were present, possibly due to loss of the drug from these areas of the surface (Figure 5F).

Overall, the results support that the acceleration in rifampicin release can be attributed to heating the coating above the *T*_g_ of PBSe. As the temperature of the PBSe coating is raised, the polymer chains become mobile, allowing the drug to diffuse through the coating to the surface, where it can then dissolve into solution. On the other hand, PBTe coatings remained below the polymer's *T*_g_, where the polymer chains are rigid.

### 2.4 Drug Release Induced by Induction Heating

A custom‐made induction heating device was developed that allowed a Ti disc to be placed in a polyethylene terephthalate glycol (PETG) sample holder such that the disc was positioned at the center of a solenoid coil. The sample holder could be filled with 5 mL of media and the temperature of the disc and media were monitored separately using a K‐type thermocouple and a thermometer, respectively. Different induction heating protocols were considered with the aim of triggering rifampicin release, while minimizing potential damage to surrounding cells and tissues. Studies have shown that the use of continuous AMF or highly hyperthermic conditions can cause necrosis of tissue, fat, or bone that surrounds the surface of the metal substrate.^[^ ^30^ ^]^ However, using an intermittent AMF has been shown to generally overcome this limitation, depending on the induction frequency and power. For example, Chopra et al. found that decreasing the exposure time but increasing power reduced the radius of thermal tissue damage.^[^ ^16c^ ^]^ Müller et al. reported that temperatures of 40–60 °C, achieved via induction heating of an intramedullary nickel‐titanium implant, resulted in no signs of bone or tissue necrosis surrounding the implant.^[^ ^18^ ^]^ Elevated temperatures are also very common in orthopedic surgery during processes such as drilling and cement fixation, but it has been suggested that bone should be kept below a critical temperature of 50 °C.^[^ ^31^ ^]^ Based on these studies, we decided to limit the surface temperature of the Ti disc to 50 °C. We found that it was possible to perform five cycles (6 min on, 6 min off) or six cycles (5 min on, 5 min off) of induction heating with a set maximum temperature of 50 °C over a period of 1 h without the surrounding bath temperature exceeding 40 °C, showing that it was possible to achieve selective heating at the metal surface while minimizing the temperature of the surrounding environment to that encountered with a common fever. After each 5–6 min heating cycle, the discs remained at 50 °C for 1–2 min, and then slowly decreased in temperature reaching around 45 °C after 3 min.

**Figure 6:**
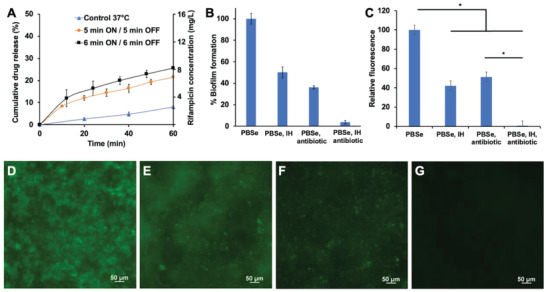
A) Release of rifampicin from inductively heated PBSe coatings (2.5% (w/w)) on Ti discs using two different heat cycling procedures, with “on” indicating heating to 50 °C and “off” indicating no induction heating (IH). The surrounding solution temperature did not exceed 40 °C. These conditions were compared to discs maintained at 37 °C with IH. Error bars correspond to the standard deviations (*n* = 3). B) *S. aureus* biofilm formation after 48 h, as measured by the crystal violet assay on PBSe‐coated Ti discs without treatment (defined as 100%) and with different combinations of antibiotic and IH. Error bars correspond to the standard deviations (*n* = 3). All treatments were significantly different from each other (*p* < 0.05). C) Relative fluorescence, based on microscopy, of biofilms formed by GFP‐expressing *S. aureus* after 48 h on PBSe‐coated Ti discs without treatment (defined as 100%) and with different combinations of antibiotic and IH. Error bars correspond to the standard deviations (*n* = 3). * indicates treatments that were significantly different statistically (*p* < 0.05). Representative fluorescence microscopy images of GFP‐expressing *S. aureus* on PBSe‐coated Ti discs with D) no treatment; E) IH; F) antibiotic; G) antibiotic and IH. In (B–G), IH treatment corresponds to the two one‐hour cycles (5 × 6 min on/6 min off) at the 2 and 24 h time points and antibiotic corresponds to the use of 2.5% (w/w) rifampicin in the PBSe coating.

As designed, induction heating of the coating above the polymer *T*_g_ accelerated drug release. PBSe coatings containing 2.5% (w/w) rifampicin released only 8% of the drug at the end of 60 min over a constant AMF at a set temperature of 37 °C (**Figure** **6**A). On the other hand, five cycles of intermittent heating with a set limit of 50 °C (6 min on, 6 min off) led to 26% rifampicin release over 60 min. Six shorter intermittent cycles at 50 °C (5 min on, 5 min off) resulted in a slower release of rifampicin with 21% released in 60 min. It appears that extending the duration of AMF can increase the release of rifampicin. It should be noted that the concentrations of rifampicin achieved in the release media (3–8 mg L^−1^) were in the range where anti‐biofilm activity against *S. aureus* has been previously observed.^[^ ^32^ ^]^ As for the drug release experiments performed with direct heating of the solution, the mechanism of accelerated drug release is proposed to involve the heating of PBSe above its *T*_g_, facilitating the diffusion of the drug to the coating surface, where it can be released. However, the key advantages of induction heating are that it can be performed remotely, using only an AMF and that the heating can be applied selectively to the metal surface, allowing temperatures well above physiological temperature to be achieved at the site of the coating (e.g., 50 °C), while maintaining the temperature of the surroundings close to normal physiological temperature.

### 2.5 Biofilm Growth and *S. aureus* Viability on Ti Discs

The abilities of the PBSe coatings to inhibit *S. aureus* proliferation and biofilm formation were investigated both with and without induction heating. *S. aureus* was selected because it is often associated with prosthetic joint infections.^[^ ^33^ ^]^ First, biofilms were grown on PBSe‐coated Ti discs in tryptic soy broth (TSB) supplemented with 1% glucose for 48 h at 37 °C and then they were quantified by crystal violet staining. The incorporation of 2.5% (w/w) of rifampicin into the PEA coating without induction heating reduced biofilm formation by 64% compared to the same PEA coating alone (Figure 6B). Induction heating alone, performed at 2 and 24 h on PBSe‐coated Ti discs resulted in a 50% reduction in biofilm formation compared to the control. Thus, neither heating nor antibiotic alone under these conditions were able to sufficiently reduce biofilm formation. However, the use of induction heating with rifampicin‐loaded PBSe coatings resulted in a 96% reduction in biofilm formation as compared to the control, showing a potential synergistic effect.

The effect of fibrinogen adsorption on the antibiofilm properties of the coatings and induction heating treatment was also investigated. Human plasma fibrinogen is a soluble glycoprotein found at high concentrations (2–3 mg mL^−1^) in human plasma. It has been reported in many studies to be among the most relevant proteins that adsorb to the surfaces of medical devices rapidly after implantation and is subsequently involved in the adhesion of cells, including platelets and leukocytes to biomaterial surfaces.^[^ ^34^ ^]^ Therefore, it was of interest to evaluate whether fibrinogen adsorption onto the PEA coating would impact the anti‐biofilm behavior of the coatings. The coatings were treated by immersion in a solution of fibrinogen, and then biofilm formation was quantified using crystal violet staining after different treatment combinations involving antibiotic and induction heating. Adsorption of fibrinogen led to a ≈20% reduction in biofilm formation compared to the PBSe surface without fibrinogen (Figure S12, Supporting Information). These results are consistent with a previous report, where the treatment of poly(ethylene terephthalate) and polytetrafluoroethylene surfaces with fibrinogen solutions led to a substantial reduction of surface adhesivity compared to uncoated surfaces.^[^ ^34a^ ^]^ While the adsorption of fibrinogen at low density has actually been shown to promote cell adhesion, at higher densities surface‐induced unfolding of the protein occurs, resulting in an extensible multilayer matrix, with weaker adhesion forces.^[^ ^35^ ^]^ Similar to the untreated PBSe coatings, the incorporation of rifampicin or application of induction heating further reduced biofilm formation, as compared to the control, by 74% and 50% respectively. Furthermore, the combination of fibrinogen, rifampicin, and heating led to a 97% reduction in biofilm formation. Overall, these results indicate that fibrinogen adsorption, expected to occur upon implantation of a PBSe coated device in vivo, should not adversely affect the coating's anti‐biofilm properties.

Biofilm studies were also performed using green fluorescent protein (GFP)‐labeled *S. aureus*. The analysis of fluorescence microscopy images showed similar trends to those observed with crystal violet staining (Figure 6C). It was evident that on the PBSe control coating, an established mature biofilm composed of GFP‐expressing bacteria had formed (Figure 6D). With induction heating alone, many GFP‐expressing bacteria were still observed, indicating that while the treatment was capable of suppressing biofilm formation to some extent, many live bacteria remained (Figure 6E). On PEA coatings containing rifampicin, but without induction heating, many GFP‐expressing bacteria were also observed (Figure 6F). Thus, neither treatment alone was sufficient. In contrast, using a rifampicin‐loaded PBSe coating in conjunction with induction heating, very few GFP‐expressing *S. aureus* colonies were observed and the relative level of fluorescence was reduced to less than 1% of the PBSe control coating (Figure 6G). Thus, these results were in good agreement with the results of the crystal violet biofilm staining and indicate that induction heating with rifamipicin‐loaded PBSe coating effectively antagonized *S. aureus* biofilm formation.

The viability of the bacteria remaining after the different treatments was also further evaluated. The biofilms were disrupted after 48 h, and the resulting suspensions were plated on agar to quantify the CFUs (**Table** **1**). This analysis revealed that induction heating or antibiotic alone reduced *S. aureus* viability, as compared to control, by 95.8 ± 0.2% and 96.3 ± 0.1% respectively. However, PEA coatings with rifampicin in combination with induction heating resulted in >99.9% (3‐log) reduction in *S. aureus* CFUs, showing that the combined treatment was much more effective than either treatment alone. Thus, despite the growth of some biofilm as indicated by the crystal violet staining, at the end of the treatment, a very small fraction of the bacteria remained viable.

**Table 1:** Quantification of viable bacteria on the coated Ti disks following different treatments (after 48 h). Standard deviations are reported for triplicate samples

Treatment	Total CFUs of *S. aureus* on the disk	% reduction in CFUs compared to untreated disk
PBSe coating, no treatment	(1.2 ± 0.1) × 10^9^	–
PBSe coating with induction heating	(5.0 ± 0.3) × 10^7^	95.8 ± 0.2
PBSe coating with antibiotic	(4.5 ± 0.2) × 10^7^	96.3 ± 0.1
PBSe coating with induction heating and antibiotic	(2.9 ± 0.3) × 10^5^	>99.9

**Figure 7:**
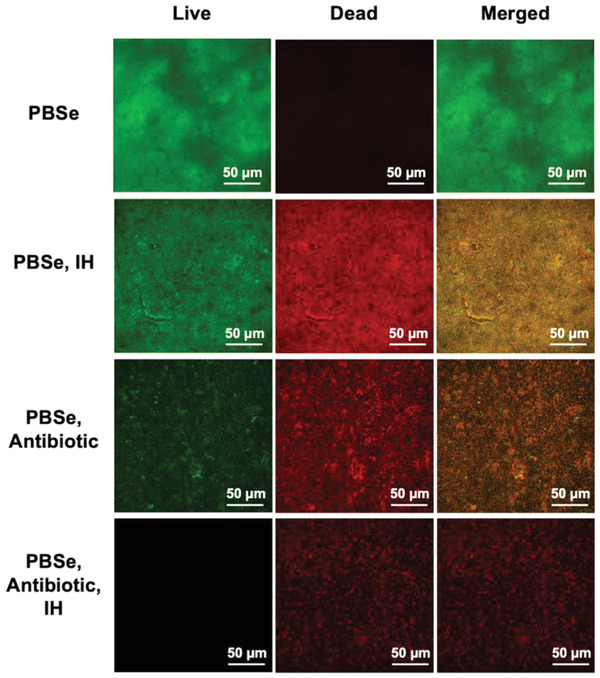
Fluorescence microscopy of *S. aureus* after LIVE/DEAD staining on PBSe‐coated Ti discs after no treatment or various treatments. IH corresponds to two 1‐h cycles of induction heating (5 × 6 min on/6 min off) at the 2 and 24 h time points and antibiotic corresponds to the use of 2.5% (w/w) rifampicin in the PBSe coating. Rifampicin‐loaded PBSe coatings treated with IH led to very few live bacteria and some dead bacteria on the surface. At least three samples were imaged for each treatment and representative images are shown.

To visualize the effects of the different treatment regimens on *S. aureus*, LIVE/DEAD staining of was also performed to distinguish viable from non‐viable bacteria (**Figure** **7**). Only live bacteria were found on the PBSe surface with no treatment. On the coating treated with two cycles of induction heating, substantial numbers of both live and dead bacteria were observed in the biofilm, confirming that induction heating had killed some but not all bacteria. A further reduction in live versus dead bacteria was observed on the rifampicin‐loaded coating without induction heating, consistent with greater suppression of biofilm formation for this treatment alone. Lastly, use of a rifampicin‐loaded PBSe coating and two cycles of induction heating yielded revealed that primarily only dead bacteria, as indicated by the presence of propidium iodide‐positive bacteria, remained on the surface. It is likely that some of these bacteria proliferated initially, but were later killed by heating and antibiotic release. The extracellular polymeric substances (EPS) produced by these bacteria may have contributed to the 3–4% biofilm (relative to control) arising from this treatment in the crystal violet assay.

The results of the bacterial studies have demonstrated that the application of a heat‐responsive antibiotic‐loaded coating can greatly enhance the effect of induction heating in killing biofilm bacteria and inhibiting the growth of biofilms on Ti materials. The approach was effective for *S. aureus*, the leading cause of orthopedic implant infection.^[^ ^8^ ^]^ Previous studies using induction heating alone have found that temperatures between 60 and 90 °C for a duration of 1–10 min were required to kill a substantial fraction of biofilm bacteria.^[^ ^16^, ^20^ ^]^ The mechanism of killing has been proposed to involve effects on bacterial cell walls and cell division, as well as a weakening of the biofilm's mechanical integrity.^[^ ^36^ ^]^ However, prolonged heating at high temperatures is not practical clinically, due to the potential for damage to surrounding tissue, limiting the efficacy of induction heating alone. Rifampicin's mechanism of action involves the inhibition of DNA‐dependent RNA polymerase.^[^ ^37^ ^]^ However, biofilm bacteria can exhibit low baseline metabolic activity, limiting the efficacy of antibiotics that inhibit protein synthesis.^[^ ^38^ ^]^ The effectiveness of the combination of antibiotic‐loaded PBSe coating and modest induction heating can be attributed to multiple factors. First, in addition to the direct killing of bacteria due to the stress of the elevated temperature, it has been found previously that heating can increase the susceptibility of biofilm bacteria to antibiotics.^[^ ^16^, ^20^, ^38^ ^]^ Such observations have been attributed to increased transport of antibiotics through the EPS as well as thermal stimulation of metabolic activity, which makes the bacteria more sensitive to antibiotics.^[^ ^38^ ^]^ Secondly, heating of the PBSe coating above the polymer's *T*_g_ increases the rate of antibiotic release from the coating, allowing it to reach inhibitory concentrations locally at the site of the biofilm. These factors combine to provide a synergistic effect. Importantly, our approach allowed us to capitalize on modest induction heating, with the Ti surface temperature not exceeding 50 °C, and the surrounding environment not exceeding 40 °C.

**Figure 8:**
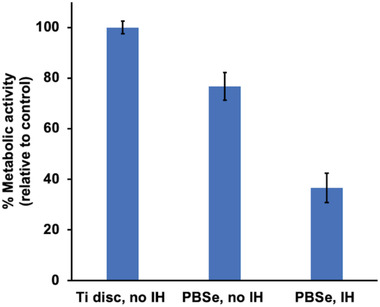
The metabolic activity of RAW 264.7 murine macrophages, as measured by an MTT assay, on uncoated Ti disks (control) and PBSe‐coated Ti disks, with or without induction heating (1 h cycle, 50 °C). All sample groups were statistically significantly different from one another (*p* < 0.05, *n* = 3).

The effects of the PBSe coating and induction heating on mammalian cells seeded directly on the polymer‐coated Ti disc were also evaluated. RAW 264.7 macrophages were selected as an initial mammalian model cell line because upon implantation of any biomaterial in vivo, these are one of the first groups of cells that interact with the foreign body and play an important role in the immunological response.^[^ ^39^ ^]^ We found that after 24 h, macrophages on the PBSe‐coated Ti disc exhibited 77% metabolic activity compared to cells seeded directly on the uncoated disc, indicating that the coating was well tolerated by the cells (**Figure** **8**). After exposure to a 1 h induction heating cycle at 50 °C, the metabolic activity of the macrophages was reduced to approximately half (37%) when compared to the PEA coating without heating. These results are in line with the known effects of elevated temperatures on mammalian cells from the field of thermal ablation therapy. It was previously reported that at 40 °C, cellular homeostasis could be maintained, while at temperatures of 46 °C for 60 min, cellular damage occurred.^[^ ^40^ ^]^ At 50–52 °C, the time to induce cytotoxicity was further decreased, and at 60 °C and higher near instantaneous protein coagulation occurred. Thus, immediately at the surface of the metal, induction heating clearly has some impact on the cells, but the presence of remaining metabolically active cells even after the treatment is promising for implant applications and suggests the potential for the cells to regenerate. Furthermore, cells not directly on the surface but in the surrounding tissue will experience much lower temperatures, similar to the 40 °C that we were able to maintain in the surrounding media. The effects of the polymer coating and IH on a suspension of red blood cells surrounding the Ti surface coated with PBSe + rifampicin were also evaluated. Over a 2 h period at 37 °C in the absence of inductive heating, negligible hemolysis was observed (0.2 ± 0.5%), indicating that the coatings themselves were very well tolerated. With a 1 h induction heating cycle (6 min on, 6 min off at 50 °C), followed by incubation at 37 °C for 1 h, hemolysis increased to 15 ± 13%. It is likely that the observed level of hemolysis can be attributed to direct contact of the cells with the surface at 50 °C, but in general these results are in agreement with the established tolerance of blood at temperatures up to 43–46 °C,^[^ ^41^ ^]^ which would reflect the conditions in the surrounding medium.

Chopra and coworkers implanted 4.8 mm stainless steel balls into the dorsal thigh muscles of mice and subjected them to induction heating for short time periods at high temperatures (100 °C).^[^ ^16c^ ^]^ The resulting tissue damage was very localized and evidence of regeneration was observed after 7 days. Müller and coworkers used electromagnetic induction to heat intramedullary nickel‐titanium implants to temperatures between 40 and 60 °C and found that there were no signs of damage based on cytokine measurements and histological analyses.^[^ ^42^ ^]^ Furthermore, temperatures close to 50 °C are routinely generated in orthopedic surgery through cortical bone drilling or polymerization of acrylic bone cement.^[^ ^31^, ^43^ ^]^ Nevertheless, the effects of induction heating on local cell populations requires careful consideration and supports the benefits of keeping temperatures moderate to support the survival of local cell populations.

Overall, our study has demonstrated the potential to achieve synergistic killing of biofilm bacteria on medical implants through the application of an antibiotic‐loaded temperature‐sensitive PEA coatings. However, some limitations and challenges remain. First, it will be necessary to further investigate the adhesion of the coating to the implant surface and its resistance to shear forces that would be encountered upon implantation into the patient. In addition, while not critical for trauma components like fixation plates, osteointegration should be investigated to determine potential applications in joint replacements. If issues are encountered with either of these aspects, we envision that the materials could be applied within the pores of porous implants; such an approach has been described in a recent study of a cellular structured hip implant prototype.^[^ ^44^ ^]^ Furthermore, it will be necessary to demonstrate the effectiveness of the coatings at eradicating infection in an in vivo model, while exhibiting acceptable biocompatibility and minimal thermal damage to surrounding tissue. Finally, to apply the approach clinically, we require a larger and more powerful inductive heating system, combined with a reliable technique to measure, or predict, the temperature of the implant and surrounding tissue. The most promising work in this regard comes from a study by Pijls et al.,^[^ ^16b^ ^]^ which investigated segmental induction heating of commonly used metal orthopedic implants to achieve surface temperatures as high as 60 °C, with thermal dose near the implant that is expected to cause little damage to soft tissue or bone.

## 3 Conclusion

PEA coatings containing rifampicin were successfully prepared on 3D‐printed Ti discs and exhibited *T*_g_ values just above physiological temperature. At normal physiological temperature, the PBSe coatings slowly eroded and released rifampicin over more than 100 days, but at temperatures above the PBSe's *T_g_* accelerated drug release was observed. Five 6‐min cycles of intermittent induction heating to a maximum of 50 °C over a period of 1 h resulted in 26% release, while the surrounding medium did not exceed 40 °C. Both heating and rifampicin individually reduced *S. aureus* biofilm formation but the use of rifampicin‐loaded PEA coatings in combination with two 1 h induction‐heating treatments over a 48 h period reduced biofilm formation by 96%. The anti‐biofilm properties were maintained even with the adsorption of fibrinogen to the surface of PEA coatings. In addition, >99.9% of the bacteria on the surface was killed during the treatment. These results were supported by fluorescence microscopy of GFP‐labeled and LIVE/DEAD staining of *S. aureus* on the Ti disc surfaces. Our results suggest that heat treatment and the thermally stimulated local release of antibiotic can perform synergistically to kill biofilm bacteria and inhibit biofilm formation. We anticipate that our new approach will provide a versatile platform that can be used to load and release different antibiotic drugs and combinations of multiple drugs. Furthermore, the amount of antibiotic should be tunable based on the drug loading of the coating as well as its thickness. These PEA‐drug blends may also be particularly useful for contemporary porous metal implant materials, as they could be loaded into pores without impacting the metal‐tissue interface. In any case, future research with these materials should focus on studying how they influence bone cell adherence, fixation of the devices, and ultimately tissue integration, along with their ability to inhibit and treat implant‐associated infection in vivo.

## 4 Experimental Section

### General Experimental Details

PBSe and PBTe were synthesized and characterized as previously reported (Figures S1–S3, Supporting Information).^[^ ^24^, ^26^ ^]^ The batch of PBSe polymer used had a number average molar mass (*M*_n_) of 79 kg mol^−1^, and a dispersity (*Đ*) of 2.5, while PBTe had an *M*_n_ of 18 kg mol^−1^ and *Đ* of 2.8. Rifampicin was purchased from Ontario Chemicals Inc. (Guelph, ON, Canada). Ti (Ti6Al4V) foil (Gr 5, ASTM F136, 0.05 mm thickness) was purchased from Shaanxi Yunzhong Metal Technology Co., Ltd. (Shaanxi, China). Dimethyl sulfoxide (DMSO) (reagent grade), dichloromethane (DCM) (glass distilled), dimethylformamide (DMF) (glass distilled) and high‐performance liquid chromatography (HPLC) grade acetonitrile (99.8%) were purchased from Caledon (Halton Hills, ON, Canada). Glacial acetic acid (reagent grade) was purchased from Caledon Laboratories Limited (Georgetown, ON, Canada). PBS (pH 7.4) was made with deionized (DI) water from a MilliQ system. Tryptic soy broth (TSB), D‐(+)‐glucose powder (bioreagent suitable for cell culture), and chloramphenicol (Cm) (Bioreagent suitable for plant cell culture) were purchased from Millipore‐Sigma (Oakville, ON) and were used as purchased without further purification. UV–visible spectroscopy was performed using a Cary 300 UV–visible spectrophotometer purchased from Varian (Palo Alto, California, USA).

### Preparation of 3D Printed Ti Discs

The 3D‐printed titanium alloy (Ti6Al4V) disks were prepared at an additive manufacturing facility (Additive Design in Surgical Solutions, London, ON) using a commercial 3D metal printer (AM400, Renishaw plc, Wotton‐under‐Edge, Gloucestershire, UK). The metal powder was consolidated using laser powder bed fusion (LPBF) with a 70 µm diameter laser spot and 40 µm layer thickness. The disks were prepared with nominal dimensions of 3 mm thickness and 24 mm diameter, with a vertical build orientation. After fabrication, the disk surfaces were smoothed using hand polishing with tungsten‐carbide burs and then finished with shot blasting, using ceramic beads (CB120, Chemco Advance Material Co., Ltd, Suzhou) with diameter ranging from 60 to 125 µm.

### Coating Preparation

Polymer‐antibiotic blends were prepared using weight percentages of 2.5%, 5%, and 10% (w/w) of rifampicin relative to the polymer. Rifampicin and the PEA were co‐dissolved in DMF at a total concentration of 16.25 mg mL^−1^ (polymer + drug), with the ratios adjusted depending on the desired percentage of drug. An aliquot of 500 µL was drop‐casted onto a 3D‐printed Ti6Al4V alloy disc (2 cm in diameter and 3 mm thick). After the solution was spread uniformly across the surface, the disc was dried in a vacuum oven at 50 °C overnight. The coating thickness (8.4 ± 1.3 µm) was measured using a digital micrometer (Mitutoyo Digital Micrometer, H‐2780, Japan) by calculating the difference between coated and non‐coated discs.

### Contact Angle Measurement

Measurements of the coatings on Ti discs were performed by the sessile drop method (10 µL of distilled water) using a goniometer (Kruss DSA100 Drop Shape Analyzer) equipped with a camera. Values were obtained after one minute of droplet incubation on the surface. Angle analysis was performed by Drop Shape Analysis software using the Laplace–Young fitting. A minimum of five water contacts angles were obtained for each coating on triplicate surfaces and the results are provided as the mean ± standard deviation.

### Scanning Electron Microscopy

SEM was performed using a Zeiss LEO 1530 instrument, operating at 2.0 kV and a working distance of 6 mm. Dried coating samples were mounted onto stubs covered in carbon tape and coated with a 10 nm layer of osmium, using an SPI Supplies, OC‐60A plasma coater. Micrographs of the surface of the coatings were taken at different magnifications.

### Thermal Analysis of Rifampicin‐PEA Blends

Rifampicin‐PEA solutions in DMF were prepared as described in the coating preparation section. The solution was then drop cast onto a glass petri dish and dried in a vacuum oven at 50 °C overnight resulting in a thin film. The films were then washed with deionized water and lyophilized. Thermal analysis was performed using a Q2000 DSC (TA Instruments, New Castle, DE, USA). The heating/cooling rate was 10 °C min^−1^ from 0 to 200 °C, and the data was obtained from the second heating cycle.

### In Vitro Release of Rifampicin

The coatings were prepared as described above. Each disc was suspended in PBS (20 mL). To measure release in the absence of thermal triggering, the samples were incubated in an oven at 37 °C for 100 d with aliquots (3 mL) of the medium, taken daily for 20 d and then every 5 d to measure the release of rifampicin. On days 20, 50, and 75 all the media was replaced with fresh PBS. To assess the effect of temperature on the rifampicin release rate, samples were also incubated in baths with set temperatures from 50–80 °C, in increments of 10 °C, for 3 h with 3 mL aliquots taken for measurement and with all the media replaced with fresh PBS every 30 min. The drug released was assessed in the PBS solution using UV–visible spectroscopy at a wavelength of 332 nm based on an extinction coefficient (*ε*) of 18 120 m ^−1^ cm^−1^ for rifampicin in the same buffer system (Figure S6, Supporting Information). Each sample and condition was studied in triplicate.

### In Vitro Degradation of Coatings in PBS

For mass loss measurements, Rifampicin‐PEA coatings (5% w/w drug) were prepared by adding 10 mL of the PEA‐rifampicin solution in DMF (30 mg mL^−1^) to pre‐weighed 5‐dram vials and then drying the coatings overnight in a vacuum oven at 50 °C. The samples were then incubated in PBS in a 37 °C oven for 105 d. Every 15 d, triplicate samples were removed, PBS was discarded, and the coatings were washed using deionized water. They were then dried to constant mass in a vacuum oven at 37 °C and then weighed to determine mass loss. To assess the effect of temperature on the degradation, the samples were incubated at set temperatures from 50–80 °C, in increments of 10 °C, for 3 h. Triplicate samples were removed every hour, washed, dried, and weighed as described above. The percent coating mass remaining was calculated as (mass at the measurement time/initial mass)*100%. Each condition was evaluated in triplicate. To image the coatings over time by SEM, coatings were prepared as described for the mass loss measurements, except that 100 µL of the PEA‐rifampicin solution in DMF was drop cast on a Ti foil square with dimensions of 1 cm × 1 cm, which was then immersed in PBS in a 5‐dram vial at 37 °C. Samples were removed every 15 days, rinsed carefully with deionized water, and imaged by SEM as described above.

### Induction Heater Design

**Figure 9:**
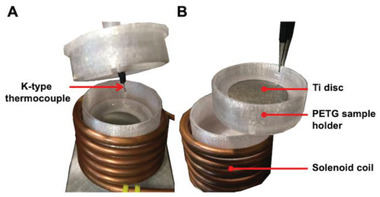
A) Induction heating device showing a solenoid coil (40 mm ID) wrapped around a 3D‐printed PETG cylinder holding in place the PETG sample holder with the K‐type thermocouple co‐axially aligned with the Ti disc sample. B) Position of the Ti disc within the PETG sample holder.

A solenoid coil (40 mm ID and 10 cm in length) and a zero‐voltage switching (ZVS) flyback transformer were developed to expose a Ti disc sample to a high‐frequency alternating magnetic field (AMF) (**Figure** **9** and Figure S11, Supporting Information). An induction frequency of 123 kHz was transmitted through the coil controlled by a dedicated micro‐controller (Arduino Uno R3). To monitor the surface temperature of the Ti disc a 3D‐printed mount was constructed to hold a K‐type thermocouple positioned in contact directly against the surface of the Ti disc and co‐axially aligned with the solenoid coil. The instantaneous power delivered to the induction heater unit was controlled in real‐time by pulse‐width modulation via a proportional‐integral‐derivative (PID) control loop. The parameters associated with the PID loop were optimized, ensuring that the desired temperature was reached within about 40 s, with minimal overshoot (±0.5 °C). Additionally, a 3D‐printed dish made from PETG was used as a sample holder for the Ti disc that could be seated at the center of the solenoid coil below the thermocouple. During induction heating experiments, instantaneous power and temperature control were monitored and recorded via Arduino software.

### Rifampicin Release Triggered by Induction Heating

For all induction heating experiments, the coatings were prepared as described in the coating preparation section. Coated discs were placed in the PETG sample holder and suspended in 5 mL of PBS with the K‐type thermocouple positioned in direct contact with the disc surface. Samples were exposed to an AMF with cycles of 6 min on and 6 min off over a period of 1 h to evaluate the relationship between AMF exposure and drug release. After various set time points, the PBS was collected for analysis and the sample was re‐suspended with new fresh PBS. The solution was analyzed for rifampicin as described in the section on in vitro release of rifampicin. A set maximum temperature of 50 °C was used for all induction heating experiments as it allowed the surrounding medium not to exceed 40 °C. This was validated using a thermometer that was placed in the media within the PETG sample holder adjacent to the sample (Figure 8). For comparison, samples were also exposed to a constant AMF at a control temperature of 37 °C. Each condition was evaluated in triplicate.

### Fibrinogen Adsorption

Fibrinogen from human plasma (50–70% protein) was purchased from Millipore‐Sigma (Oakville, ON), aliquoted, and stored at −20 °C. Before coating, the required aliquots were thawed, diluted to 25 µg mL^−1^ in 0.9% (w/v) NaCl, and sterilized by filtration through a 0.22 µm low binding protein syringe filter. Ti discs were placed in six‐well plates and coated with fibrinogen via immersion in 3 mL of the fibrinogen solution for 30 min at 37 °C. Then, the discs were removed and dried at room temperature overnight in a glass petri dish.

### General Bacterial Culture and Biofilm Growth Procedures

*S. aureus* (ATCC 6538) was grown using a tryptic soy agar (TSA) plate incubated for 18 h at 37 °C, followed by the inoculation of a single colony in TSB for culture. The *S. aureus* was maintained over the long term in a glycerol stock incubated at ‐80 °C. Coatings were prepared as described in the coating preparation section. The sample groups included PBSe coatings without induction heating or rifampicin, PBSe coatings with induction heating and no rifampicin, rifampicin‐loaded PBSe coatings with no induction heating, and rifampicin‐loaded PBSe coatings with induction heating. Biofilms were grown in TSB supplemented with 1% (w/v) glucose. This medium was inoculated with *S. aureus* using an inoculation loop and incubated for 24 h at 37 ± 0.5 °C. The inoculated medium was diluted to a bacterial concentration of approximately 10^8^ colony‐forming units per mL (CFU mL^−1^), validated with the optical density of the stock measured at 600 nm using a spectrophotometer. The stock was further diluted with sterile TSB + 1% (w/v) glucose to give a working bacterial concentration of ≈10^5^ CFU mL^−1^. Coated or uncoated Ti discs were sterilized using the biosafety cabinet UV light for 1 h on each side and then were immersed the inoculated medium (10 mL) for 2 h, allowing for bacterial adherence. Afterward, the excess medium was discarded, and discs were incubated in 10 mL of fresh medium for 48 h at 37 °C.

### Measurement of *S. aureus* Biofilm Growth and Bacterial Viability

*S. aureus* was cultured, suspended, and the sample surfaces were immersed in the suspension as described in the preceding section. Immediately after the 2 h incubation period to allow for bacterial adherence, discs were transferred to the PETG sample holder and immersed in 5 mL of fresh TSB and 1% (w/v) glucose. Samples were then exposed to an AMF intermittently, 6 min on and off, for a total period of 60 min. A maximum set temperature of 50 °C was used to prevent the surrounding medium in the sample holder from exceeding 40 °C. Upon completion of intermittent AMF exposure, the discs and 5 mL of media were transferred into a new small polystyrene petri dish containing 5 mL of fresh medium for an added total of 10 mL and then incubated for 24 h at 37 °C. After 24 h, the disc and 5 mL of media were again transferred to the PETG sample holder, the induction heating process was repeated as above, and then the disc and media were transferred back to the petri dish and incubated for an additional 24 h.

Biofilm formation was assessed using crystal violet (CV) staining.^[^ ^45^ ^]^ After the incubation period, excess media was removed, and the discs were gently rinsed in fresh PBS three times. The biofilms were fixed by incubation for 1 h at 60 °C and then stained by immersion of the discs in 5 mL of 0.1% (w/v) CV for 2 h in a 6‐well polystyrene plate. The excess stain solution was removed, and the samples were gently rinsed with sterile distilled water. The stained biofilms were then destained using 33% (v/v) acetic acid and the optical density of the acetic acid solution was measured at 595 nm using a Tecan Infinite M1000 Pro plate reader using Costar 96‐well UV plates (#3635) with UV transparent flat bottoms. Each treatment group was evaluated in triplicate and the results were normalized to the PBSe coating without IH or rifampicin as 100% biofilm.

The viability of the *S. aureus* after the different treatments was also quantified. After the completion of each treatment, the Ti discs were placed in 50 mL centrifuge tubes and the established biofilms were disrupted using a Fisher Scientific Ultrasonic Bath FS20H (Hampton, NH, USA) for 20 min at 40 kHz. The Ti discs were removed and then the sonicated suspension was centrifuged for 15 min at 5000 rpm (2900 g) in a VWR Clinical 200 Centrifuge (Radnor, PA, USA). The suspension was subsequently vortexed and then serially diluted tenfold into 15 mL centrifuge tubes. 10 µL of each dilution were spot plated onto TSA plates and allowed to absorb for 30 min. The TSA plates were then inverted and incubated at 37 °C for 24 h. The plates prepared from dilutions that resulted in 3–50 CFUs were counted and after taking dilutions into account the CFU reduction for each treatment was calculated. Each treatment was analyzed in triplicate.

### Fluorescence Microscopy of GFP‐Labeled *S. aureus* on Coated Discs

To visualize the effects of rifampicin‐PEA coatings with and without IH on *S. aureus* biofilms, GFP expressing *S. aureus* was used. The *S. aureus* (ACC 6538) strain was transformed by electroporation with the plasmid pCG44 to enable GFP expression.^[^ ^46^ ^]^ The plasmid was maintained in *S. aureus* by culture in the presence of Cm at 10 µg mL^−1^. TSB + 1% (w/v) glucose + Cm were inoculated with the GFP‐labeled *S. aureus* for 24 h at 37 °C. The inoculated medium was then diluted to a working bacterial concentration of about 10^5^ CFU mL^−1^. Discs were then treated as described in the preceding section. After the final 24 h incubation, the discs were gently rinsed with PBS and then imaged using a Nikon Eclipse Ti2E Inverted Deconvolution Microscope from Nikon Instruments Canada Inc. (Mississauga, ON, Canada) at 20× magnification. The excitation and emission wavelengths were 475 and 515 nm respectively. To compare the levels of fluorescence in the different images, the optical density was integrated across all pixels (all images had the same number of total pixels) using Image‐Pro 10 software (Media Cybernetics Inc., Rockville, Maryland, USA) and then the data was normalized to the PBSe control coating, which was defined as 100%. Three different images were analyzed for each treatment.

### Live/Dead Analysis of *S. aureus* on Coated Discs

To visualize the viability of *S. aureus* (not GFP‐labeled) in the biofilms, the discs were treated as described in the preceding sections. After the final 24 h incubation period, the bacteria were stained using a LIVE/DEAD BacLight Bacterial Viability Kit (L7007, Thermofisher Scientific, Waltham, MA, USA) with propidium iodide (PI) to detect dead cell populations and SYTO 9 to detect viable cells. The stain was prepared according to the manufacturer's instructions and added directly onto the surfaces of all samples. It was then incubated at room temperature for 10 min in the dark. Imaging was performed using a Nikon Eclipse Ti2E Inverted Deconvolution Microscope from Nikon Instruments Canada Inc. (Mississauga, ON, Canada) at 20× magnification using excitation/emission wavelengths were 485/585 for propidium iodide (red stain) and 485/530 nm for SYTO‐9 (green stain). To establish the microscope settings, bacteria on antibiotic free and non‐heated PBSe coatings were used as live bacteria controls, while biofilms killed by heat shock treatment at 150 °C for 10 min prior to staining were used as dead cell controls. All coatings were then imaged using the same microscope settings.

### Mammalian Cell Culture

RAW 264.7 (ATCC TIB‐71TM) immortalized murine macrophages were cultured in Dulbecco's Modified Eagle Medium (DMEM) containing 10% fetal bovine serum (FBS) and supplemented with antibiotics penicillin and streptomycin, 100 units mL^−1^ each (Life Technologies, Burlington, Canada) at 37 °C in the presence of 5% CO_2_. Ti discs coated with only PBSe as described in the coating preparation section and uncoated Ti discs were sterilized by irradiation using a biosafety cabinet UV light for 1 h on each side. Cells were then seeded at a density of 184 000 cells (300 µL) onto each Ti disc in a six‐well polystyrene plate and incubated for 3 h to allow initial adherence. An additional 3 mL of culture media was then added to each well and samples were incubated for 24 h. After 24 h, PBSe‐coated Ti discs were subjected to a 1 h IH protocol (alternating 6 min on, 6 min off at a set maximum temperature of 50 °C). Upon completion of the IH cycle, the medium was aspirated and replaced with 3 mL of culture media containing 0.5 mg mL^−1^ of MTT and the cells were incubated for 3 h. The MTT solution was then carefully aspirated, and 1 mL of DMSO was added to each well to solubilize the purple crystals. The absorbance of each well at 570 nm was then read in a plate reader (Tecan Infinite M1000 Pro) to quantify the relative metabolic activities. Triplicates of all treatments were performed.

### Red blood Cell Hemolysis

Whole human blood was obtained in compliance with the Office of Research Ethics at The University of Western Ontario (protocol 109059). ≈20 mL of whole blood was collected from healthy volunteers and was separated using lympholyte‐poly (Cedarlane Laboratories) according to the manufacturer's instructions to isolate human red blood cells (hRBCs). Pelleted hRBCs were resuspended in 30 mL of sterile PBS and stored at 4 °C for use within 5 days post‐isolation. Upon use, hRBCs were pelleted by centrifuging 1.7 mL of blood and washing the pellet four times with 0.9% saline. The pellet was resuspended in PBS (pH 7.4, 1.7 mL) to create the hRBC suspension. A negative control (defining 0% lysis) was prepared by adding 50 µL of the hRBC suspension to 2 mL of PBS, while a positive control defining 100% lysis) was prepared by adding 50 µL of the hRBC suspension to 2 mL of deionized water. Then, a diluted hRBC suspension for addition to the coated discs was prepared by adding 1.0 mL of the hRBC suspension to 40 mL of PBS. PBSe coatings containing 5% (w/w) of rifampicin were prepared by drop casting 500 µL of a DMF solution of the polymer + drug (16.25 mg mL^−1^) onto a Ti foil (18 mm diameter). The foil was then placed on a steel core disc (18 mm diameter, 1.2 mm thickness) and the disc was immersed in 4 mL of the diluted hRBC suspension. To evaluate the effect of IH, discs in the RBC suspension were subjected to a 1 h IH protocol (alternating 6 min on, 6 min off at a set maximum temperature of 50 °C) followed by incubation at 37 °C for 1 h, while another set, along with the positive and negative controls, were incubated at 37 °C for 2 h. Afterward, the hRBC suspensions were removed and centrifuged to re‐pelletize the blood cells. 200 µL of the supernatant of each sample and control were placed into wells in a 96 well plate and the absorbance at 575 nm was measured on a M1000‐Pro plate reader (Tecan, Mannedorf, Switzerland). The percent hemolysis was determined using Equation (1):

(1) $$\mathrm{Percent}\;\mathrm{Hemolysis}\;=\;\frac{A_{\mathrm{sample}}\;-\;A_{\mathrm{negative}\;\mathrm{control}}}{A_{\mathrm{positive}\;\mathrm{control}\;}-\;A_{\mathrm{negative}\;\mathrm{control}}}\times 100\%$$

### Statistical Analysis

For Figure 6B,C the data were normalized to the PBSe coating without IH or rifampicin as 100% biofilm and 100% relative fluorescence respectively. For Figure 8, the data were normalized to an uncoated Ti disc without heating as 100% metabolic activity. All data are presented as the mean ± standard deviation and all treatments across the different experiments were repeated in triplicate (*n* = 3). Statistical analyses were performed using one‐way ANOVA with Tukey's post‐hoc tests (Figures 6, 8, Figure S12) using GraphPad Prism. Differences were considered statistically significant at *p* < 0.05.

## Conflict of Interest

The authors declare no conflict of interest.

## Supporting information

Supporting Information

## Data Availability

The data that support the findings of this study are available from the corresponding author upon reasonable request.
