# Supplementary material for: Induction Heating Triggers Antibiotic Release and Synergistic Bacterial Killing on Polymer‐Coated Titanium Surfaces
Source: Adv Healthc Mater. 2023 May 1;12(22):2202807. doi: 10.1002/adhm.202202807 (PMC11469058; doi:10.1002/adhm.202202807)
Supplement: Supplementary file 1 — Supporting Information [file ADHM-12-2202807-s001.pdf]

ADVANCED HEALTHCARE MATERIALS

Supporting Information

for *Adv. Healthcare Mater.*, DOI 10.1002/adhm.202202807

Induction Heating Triggers Antibiotic Release and Synergistic Bacterial Killing on Polymer-Coated Titanium Surfaces

*Jan C. Kwan, Ronald S. Flannagan, Mónica Vásquez Peña, David E. Heinrichs, David W. Holdsworth and Elizabeth R. Gillies**

Supporting information for:

Induction heating triggers antibiotic release and synergistic bacterial killing on polymer-coated titanium surfaces

Jan C. Kwan, Ronald S. Flannagan, Mónica Vásquez Peña, David E. Heinrichs, David W. Holdsworth, and Elizabeth R. Gillies*

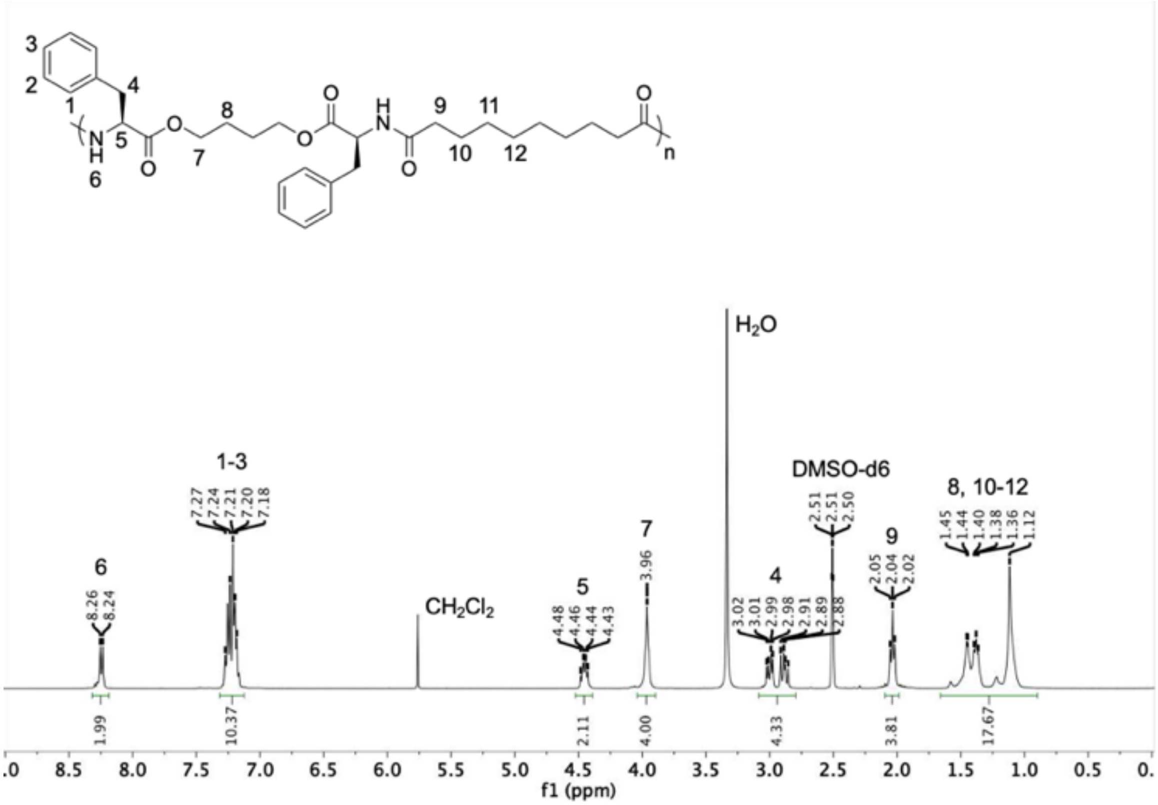

Figure S1. ^{1}H NMR spectrum of PBSe (DMSO-d_6, 400 MHz).

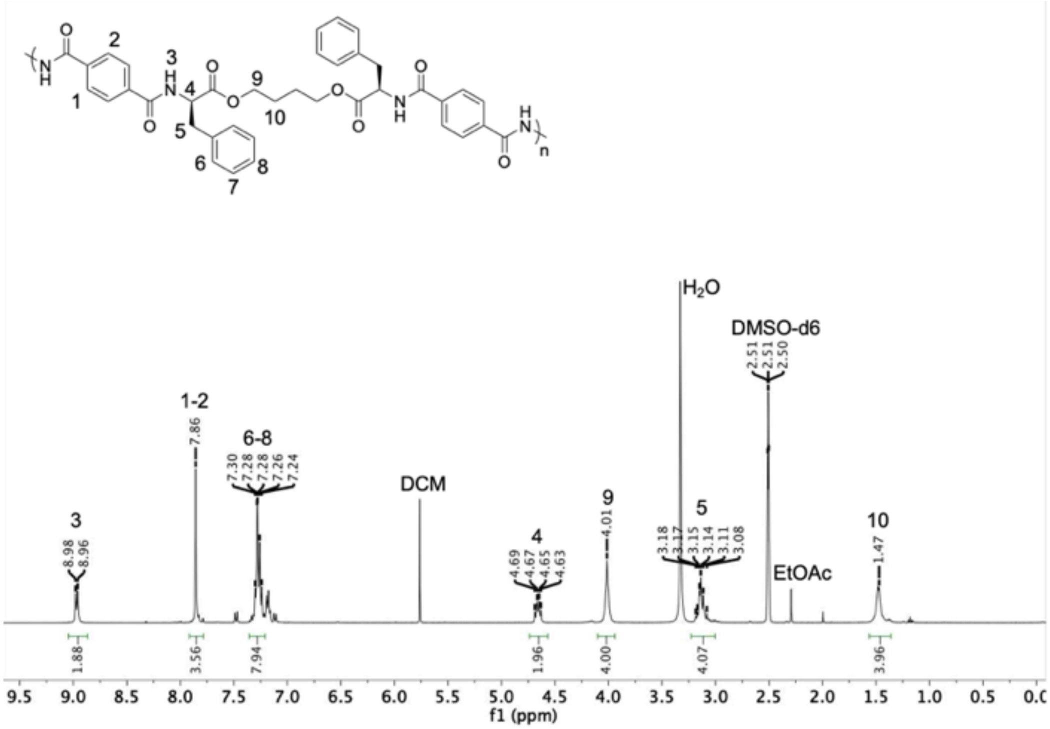

Figure S2. ^{1}H NMR spectrum of control polymer PBTe (DMSO-d_6, 400 MHz)

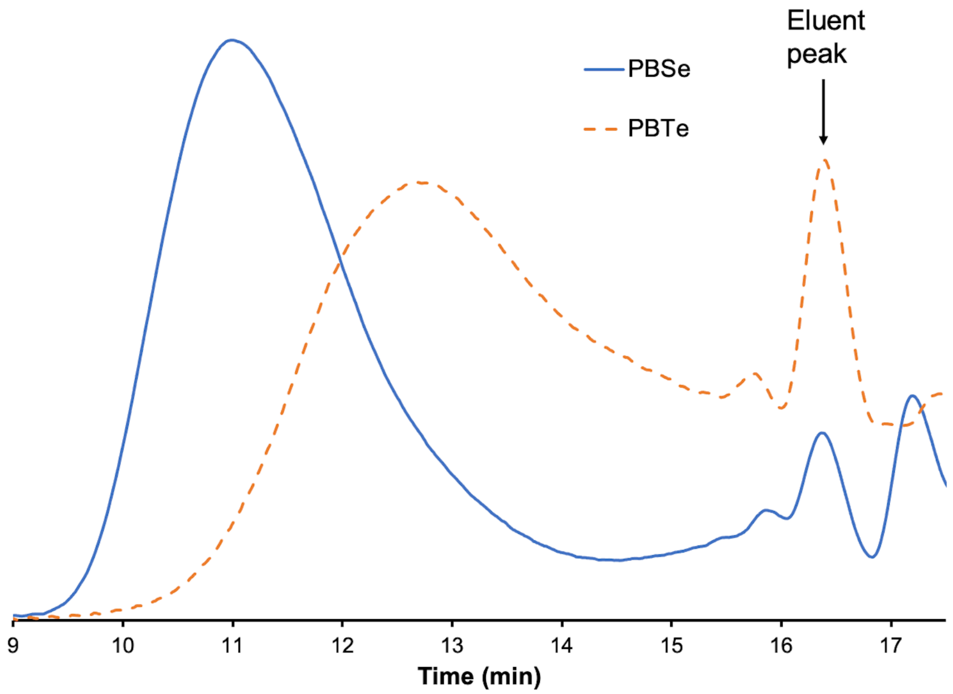

Figure S3. Overlay of SEC traces for PBSe and PBTe (detection by relative refractive index).

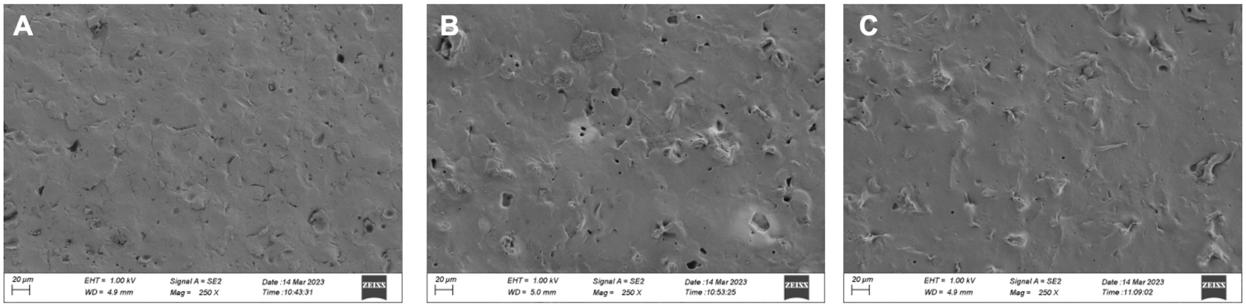

Figure S4. SEM images of A) Initial 3D printed Ti disc; B) PBSe coated Ti disc; C) PBSe + 5% rifampicin coated Ti disc.

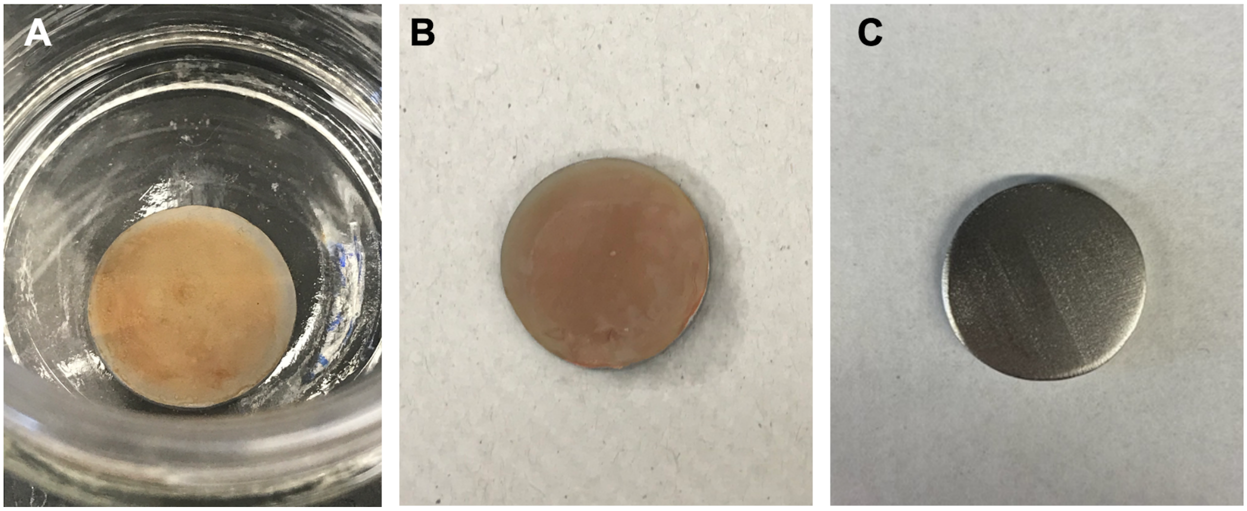

Figure S5. Digital images of: A) PBSe + 5% rifampicin coated Ti disc still immersed in PBS after 105 days; B) PBSe + 5% rifampicin coated Ti disc after removal from PBS after 105 days. The presence of coating on the disc can be visualized by its reddish colour in contrast to C) an uncoated Ti disc.

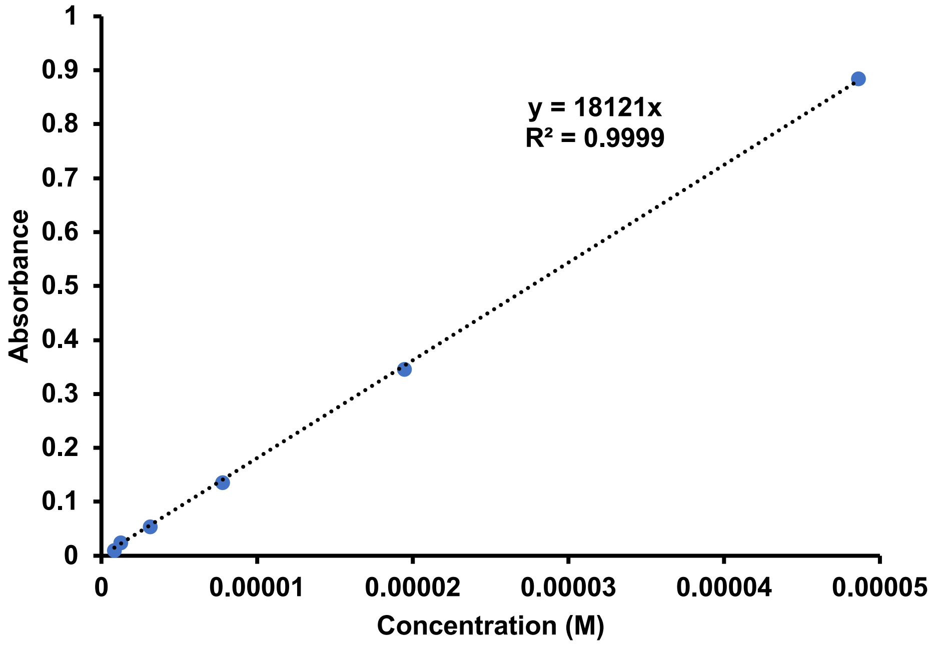

Figure S6. Calibration curve for rifampicin at 332 nm, obtained by UV-visible spectroscopy. The rifampicin solutions were obtained by serial 5-fold dilutions in PBS.

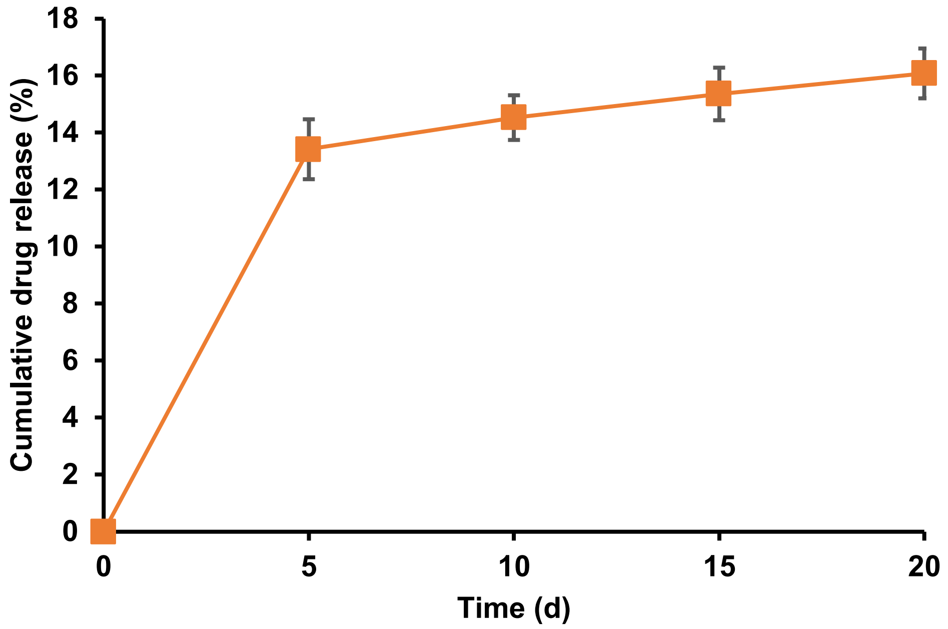

Figure S7. Release of rifampicin from PBSe coatings containing 5% (w/w) rifampicin at 37 °C in pH 5.0 acetate buffer (100 mM). Error bars correspond to the standard deviations on five samples. The experiment was performed using the same procedure as for the study in pH 7.4 PBS, with only a change in the buffer, length of the experiment (20 days), and ε (27260 $M^{-1}cm^{-1}$) in the pH 5.0 buffer.

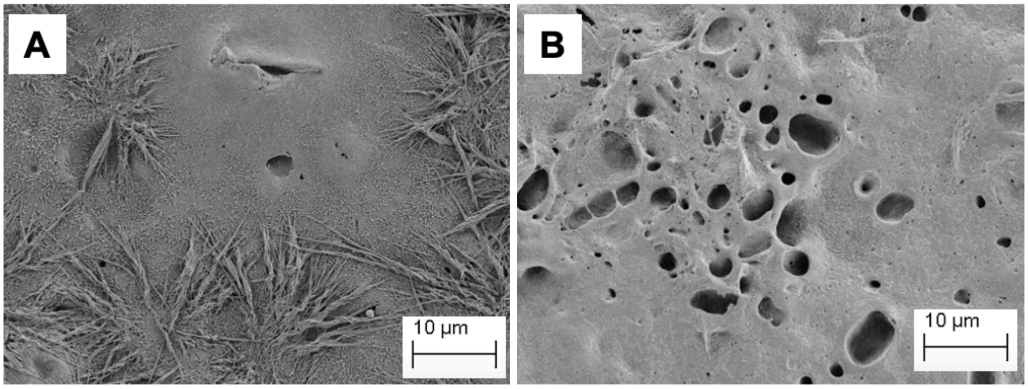

Figure S8. Scanning electron micrographs of PBSe coatings containing 5% (w/w) rifampicin after incubation in PBS at 37 °C for A) 15 days and B) 90 days.

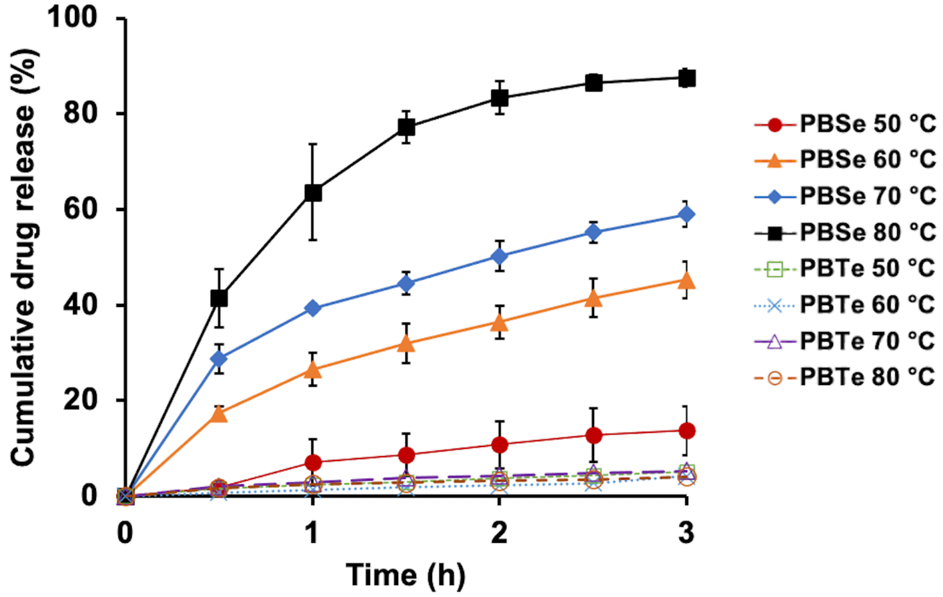

Figure S9. Release of rifampicin from PBSe and PBTe coatings containing 10% (w/w) rifampicin at varying temperatures in PBS. More rapid release of drug occurred for the PBSe coatings but not for the PBTe coatings at elevated temperatures. Error bars correspond to the standard deviations on triplicate samples.

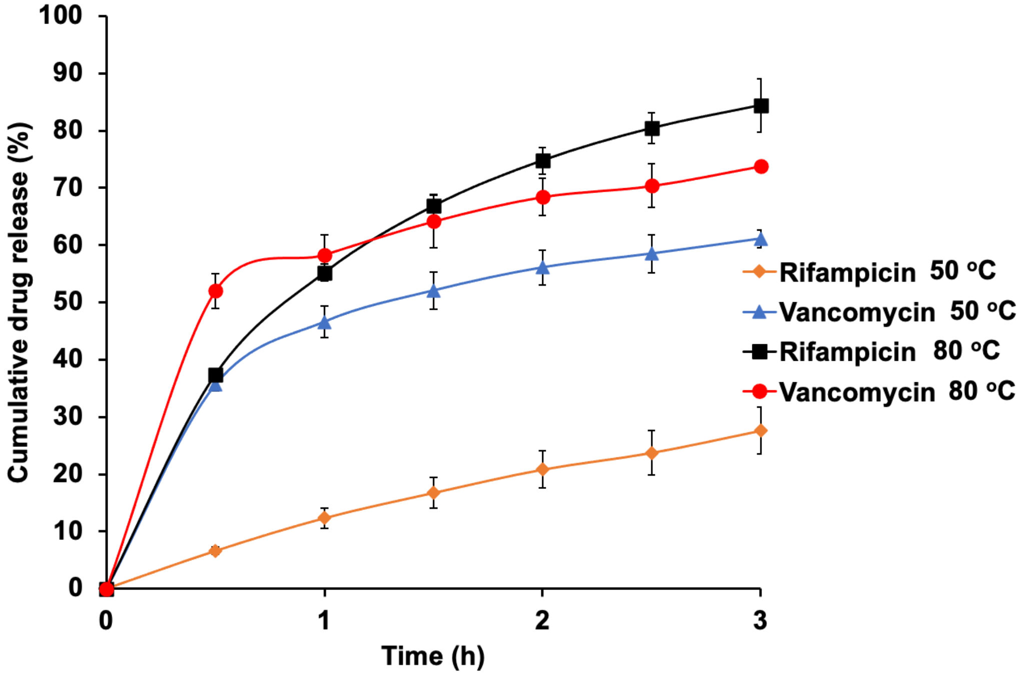

Figure S10. Release of rifampicin and vancomycin from PBSe coatings containing 5% (w/w) of each drug at varying temperatures in PBS. These coatings were prepared as described in the "Coating preparation" section of the experimental procedures except that 5% (w/w) vancomycin was included in addition to 5% (w/w) rifampicin. Rifampicin release was measured first based on the absorbance of the release media at 332 nm as described in the "In vitro release of rifampicin" section of the experimental procedures. Vancomycin release was measured by the same procedure as rifampicin but was based on the absorbance at 280 nm (ε = 6380 M^{-1} cm^{-1}) after first subtracting the corresponding absorbance of rifampicin at this wavelength. Error bars correspond to the standard deviations on triplicate samples.

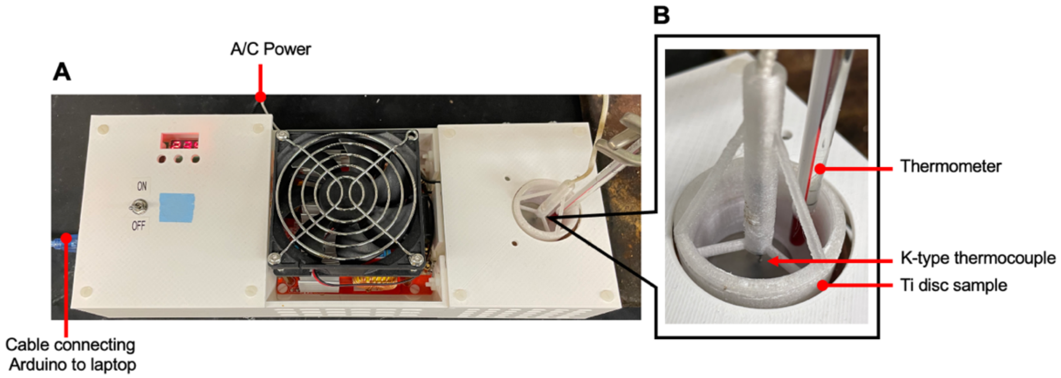

Figure S11. A) Top view of the custom-made inductive heating device and B) Expanded view of a 3D-printed Ti disc sample at the center of a PETG sample holder with a K-type thermocouple in contact with the surface of the Ti disc and thermometer to measure the surrounding medium temperature.

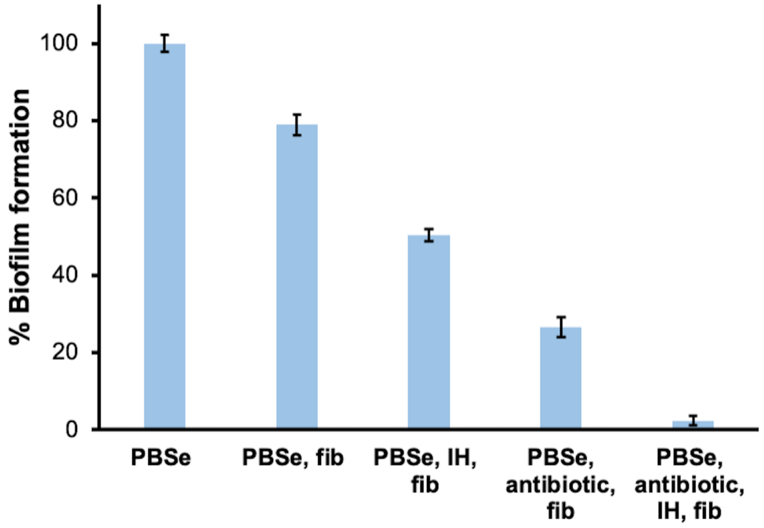

Figure S12. *S. aureus* biofilm formation as measured by the crystal violet assay on untreated PBSe coatings (defined as 100%) and PBSe coatings treated first with fibrinogen, followed by different combinations of antibiotic and IH. Error bars correspond to the standard deviations on triplicate samples. All samples were statically significantly different from one another ($p < 0.05$).
